# Coagulation Factor IX for Hemophilia B Therapy 

**Published:** 2012

**Authors:** N. A. Orlova, S. V. Kovnir, I. I. Vorobiev, A.G. Gabibov

**Affiliations:** Shemyakin and Ovchinnikov Institute of Bioorganic Chemistry, Russian Academy of Sciences; Hematology Research Centre, Ministry of Healthcare and Social Development of the Russian Federation

**Keywords:** сoagulation factor IX, hemophilia B, heterologous protein expression systems

## Abstract

Factor IX is a zymogen enzyme of the blood coagulation cascade. Inherited absence or deficit of the IX functional factor causes bleeding disorder hemophilia B, which requires constant protein replacement therapy. Reviewed herein are the current state in the manufacturing of FIX, improved variants of the recombinant protein for therapy, transgenic organisms for obtaining FIX, and the advances in the gene therapy of hemophilia B.

## Introduction 

**Fig. 1:**
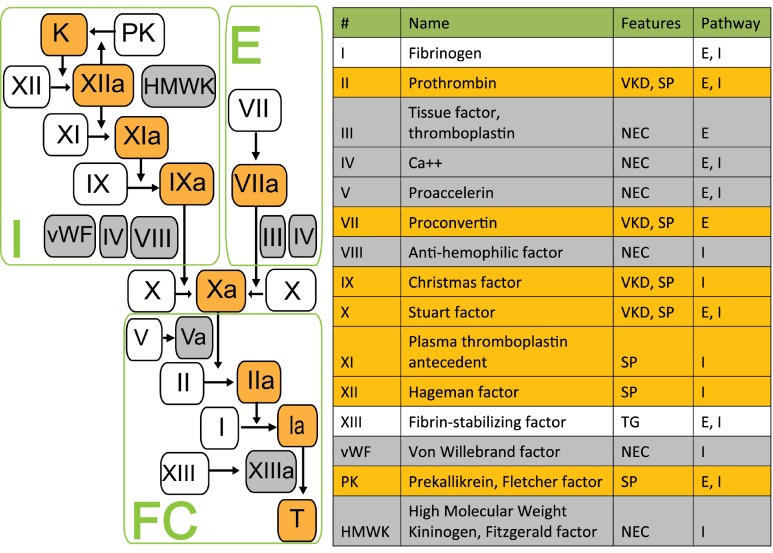
Scheme of blood coagulation cascade and international nomenclature of coagulation factors [2]. *VKD - vitamin K dependent; SP - serine protease, TG - transglutaminase; E – extrinsic pathway; I – intrinsic pathway; NEC - non-enzymatic cofactor, FC - final common pathway. *

Factor IX (FIX, Christmas factor) is a blood clotting factor, a zymogen of serine protease. Upon activation, FIX is converted into the active serine protease and, in the presence of Ca ^2+^ and membrane phospholipids, it hydrolyses one arginine-isoleucine bond in factor X to form the activated factor X (Xa) [1]. The catalytic efficiency of activated FIX (FIXa) is greatly increased by the cofactor, the activated factor VIII (FVIIIa). The non-covalent complex of FIXa, FVIIIa and FX, bound to the phospholipid membrane, is called “the X-ase” or “tenase” and represents a major signal amplification loop in the blood coagulation cascade ( *Fig. 1* ). 

Factor IX is produced in the liver, and the inactive precursor protein is processed in the endoplasmic reticulum and Golgi, where it undergoes multiple post-translational modifications and is secreted into the bloodstream upon proteolytic cleavage of the propeptide. Circulated mature FIX, 57 kDa and app. 90 nM, takes part in the blood coagulation cascade after specific proteolytic cleavage by the activated factor XI (of the contact pathway) or the activated factor VII (of the tissue factor pathway), with the formation of two polypeptide chains linked by a single disulfide bridge. Activated FIX is slowly deactivated by multiple factors – binding to antithrombin III, nexin-2, the protein Z-dependent protease inhibitor, and endocytic hepatocyte receptors or degraded by neutrophil elastase [3].  

The gene of human FIX lies in the X chromosome, has 8 exons, and spans 33.5 Kb. Various mutations in this gene can impair the functioning of the FIX protein, resulting in bleeding-disorder hemophilia B: these mutations are present in the dedicated database [4]. The rate of incidence of severe hemophilia B, requiring regular replacement therapy, is 1 in every 30,000 men, which represents approximately 20% of all hemophiliacs. Recently, it has been proved that European royalty suffered from hemophilia B: the last affected person passed away in 1940 [5]. The point mutation discovered in these kindred resulted in altered splicing and truncated form of the FIX protein.  

In some cases, mutations in the promoter region of the gene result in the less severe hemophilia B Leiden [6], characterized as a nearly complete absence of FIX in childhood and steady increase in the level of endogenous FIX during puberty to the near-normal values. 

Current treatment of hemophilia B is restricted to protein-replacement therapy, which is very expensive for patients and the healthcare system. Only 20% of the world’s population can afford the treatment; so hemophilia B remains lethal in childhood in poor countries [7].  

## Replacement Therapy of Hemophilia B 

Initial specific therapy of hemophilia B used to consist of periodic treatment by plasma transfusions, later replaced by more effective prothrombin complex concentrates (PCC), which contain a mixture of VKD pro-coagulation factors, including FII, FVII and FX. The most significant drawback of PCC is the risk of thrombotic episodes. Purer preparations of FIXhave been isolated through Cohn fractionation by ion-exchange chromatography. The safety of plasma-derived FIX preparations has also been improved by the introduction of various virus-inactivation steps, including heating, thiocyanate, and solvent-detergent treatment, which allow to remove enveloped viruses; and nanofiltration, to remove nonenveloped viruses [8]. 

Another limitation on the safety of FIX plasma concentrates is placed by the detectable amount of activated FIX (FIXa) and residual levels of other pro-coagulation factors, which result in a still significant risk of thrombotic episodes. Immunoaffinity purification of plasma-derived FIX has been sufficient to overcome these limitations [9], but as in any other plasma-derived product, the risk of viral and prion transmission remains [10].  

## Recombinant FIX 

Cloning of FIX cDNA was reported in 1982 [11], and biologically active FIX was expressed in a rat hepatoma cell line, mouse fibroblasts, and baby hamster kidney (BHK) cells in 1985 [12-14]. Expression of FIX in industrially suitable CHO cells was achieved in 1986 [15].  

The first and only marketed medicinal product of recombinant FIX to date is Nonacog alpha (trade name Benefix). It was approved for clinical use in the U.S. and European Union in 1997. Nonacog alpha is expressed by CHO cells, cultivated in an animal origin components-free medium, purified through 4 chromatographic steps without the use of immunoaffinity columns, and virus-inactivated by nanofiltration with a cut-off limit of 70 kDa [16]. The final product is formulated as a lyophilized powder without human serum albumin [17]. Initial formulation allowed for 250- to1,000-IU strength in one vial, and reformulation of recombinant FIX extended this interval up to 2000 IU/vial strength [18], additionally allowing for room-temperature storage. 

**Fig. 2:**
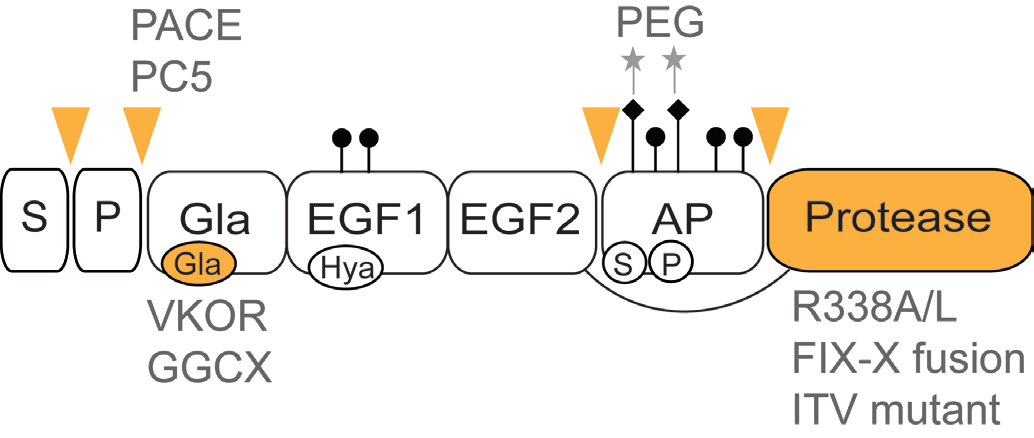
Structure of FIX. S – signal peptide; P – propeptide, Gla – Gla domain, EGF1 and EGF2 – epidermal growth factor-like domains, AP – activation peptide, Protease – serine protease domain. Posttranslational modifications sites: Gla – γ-carboxylation, Hya – β-hydroxylation; S – sulfation, P – phosphorylation, ♦ – N-linked and ● – O-linked glycosylation, cleavage points are indicated by triangles, main possible routes for factor IX production or activity improvement are typed in grey.

Clinical studies of recombinant FIX have showed that the safety and efficacy of recombinant and plasma-derived FIX are comparable, and no evidence of viral transmission was detected after 1,514 infusions of recombinant FIX to 56 patients [19]. The immune response level to infused recombinant FIX was also comparable to plasma-derived FIX [20].  

Recombinant FIX has been extensively investigated for structural deviations from natural FIX and the significance of such deviations. The germinal studies claimed that there were close similarity in post-translational modifications [21], although the adjusted recovery of FIX activity following a bolus infusion was found to be significantly lower for recombinant FIX [22], resulting in a 1.5- to 2-fold increase of the recommended dose [23]. The pattern of post-translational modifications was found to be indistinguishable between lots of recombinant FIX from two production plants[24]; thus, the differences in recombinant and plasma-derived FIX appear to be rather process-specific. 

## Structure and post-translational modifications of FIX 

The FIX protein is a member of vitamin K-dependent (VKD) blood clotting factors and consists of four structural domains: the Gla domain, two tandem EGF-like domains, and a C-terminal serine protease domain ( *Fig. 2* ). The N-terminal signal peptide of FIX is released after translocation to endoplasmic reticulum, and the propeptide, immediately preceding the Gla-domain, is removed at secretion. The activation peptide, which is located between the second EGF domain and the serine protease domain, is specifically cleaved by the factor XIa at the activation of FIX. 

The N-terminal Gla domain of mature FIX, common to members of the VKD group, mediates the binding of FIX and FIXa to the surface of endothelial cells; this interaction is disrupted completely if γ-carboxylation of the Asp residues in the Gla domain is blocked [25]. The first EGF-like module is known to have a high-affinity Ca ^2+^ binding site and to take part in the interaction with factor VIIIa [26] and with the tissue factor [1]. It contains an unusual modification of the Asp64 residue to β-hydroxyaspartate (Hya). This modification does not affect the pro-coagulation properties of FIX [25], but exchange of the Asp64 residue to a basic or neutral amino acid impairs the activity of FIX [27]. Second EGF-like module also participates in the assembly of the FIXa-FVIIIa-FX complex [28, 29]. The EGF2 and protease domains of FIX are connected via the activation peptide and a single disulphide bond.  

The activation peptide contains many sites of post-translational modifications that have various impacts on the properties of the protein (summarized in *Table 1* ). The protease domain accounts for half of the mass of FIX. The serine protease active site in this domain is buried under the activation peptide and exposed after its cleavage. There are no known post-translational modifications in this domain; upon activation, it connects to the rest of the FIX molecule by a single disulphide bond located opposite the active site. The C-terminus of the activated FIX also lies far from the active site cavity of protease and allows the creation of functionally active fusion protein molecules.  

**Table 1:** Post-translational modifications of FIX and its derivatives

Structural feature	Domain	Function of PTM	pdFIX	rFIX (BeneFix)	Fc fusion	PEG conjugate
Gamma-carboxilation (Glu → Gla)Total Gla and Gla share	Gla	Interaction with membrane, binding of Ca^2+^	total 12 (12/12)	total 11,6 (60% 12/12, 35%11/12, 5% 10/12)	Ttl 11,2	total 11,6 (33% 11/12; 64% 12/12)
beta-hydroxylation (Asp64 → Hya)	EGF	ND	37%	46% - 49%	70%	partial
N-linked glycans	AP	ND	3- and 4-antennary, sialated by Neu-5-Ac	more complex structure than pd, contain different linkages, more fucosilation and poly-acetillactoseamine structures		Both present, fucosylated core, 3- and 4-antennary, complex type
Asn157 heterogeneity	AP		high	low	low	sialated*in vitro*
Asn167 sialation	AP		Full	less	not full	
O-linked glycans					different from CHO-derived FIX, relative ratios	
Ser 53	GLA	ND	(Xyl)1-2-Glc	(Xyl)2-Glc		Present
Ser 61	GLA	ND	NeuAcGalGlcNAcFuc	NeuAcGalGlcNAcFuc		Present
Thr 159, Thr 167, Thr 172, Thr179 (?)	AP	Blocks protease active site	partial	partial		Partial
Tyr 155 sulfation	AP	Accounts for*in vivo*activity recovery	>90%	5%-15%	4%	ND
Ser 158 phosphorylation	AP	Accounts for*in vivo*activity recovery?	>90%	<10%	<10%	ND
Activated FIX		Unwanted admixture	0.21%+_ 0.010%	0.11+-0.0019%	<0.013%	0.03%

Although the only post-translational modification with a direct and significant effect on the pro-coagulation activity of FIX is the γ-carboxylation in the Gla domain [30], other modifications also play a more or less clear role in the functioning of FIX.  

The decrease in activity recovery in recombinant FIX was assigned by the research group of the Genetics Institute, Inc., to two PTM’s – absent phosphorylation of Ser158 and nearly absent sulfation of Tyr 155 in the recombinant FIX [21]. It was demonstrated that infusion of recombinant FIX enriched in the sulfated variant results in increased activity recovery, and, at the same time, that isolation of recombinant FIX from infused hemophilia B dogs results in the enrichment of the material by the sulfated FIX. It should be noted that both sites of the modification are located in the activation peptide and are in very close proximity to each other, as well as to the N-linked glycan at Asn157, making the separation of sulfated and phosphorylated molecules questionable.  

Natural FIX undergoes both O- and N-glycosylation. Berthing sites of O-linked oligosaccharides are present in the EGF1 domain [31] and activation peptide. Two O-linked oligosaccharides in the EGF domain are present uniformly in natural and recombinant FIX, and 2 to 4 possible O-glycosylation sites in the activation peptide are modified only partially [32, 33].

There are also two sites for the mooring of N-linked oligosaccharides in the activation peptide of factor IX at the asparagine residues 157 and 167 [11]: both sites are fully occupied by predominantly sialated oligosaccharide groups in plasma-derived FIX [34]. Enzymatic cleavage of all sialic acid residues from the O- and N-linked groups does not alter the activation rate of FIX and its ability to activate factor X [35]. At the same time, the low level of sialation in N-linked glycans in the recombinant FIX may account for the differences in the binding to endothelial cells, the rate of clearance from circulation, or the susceptibility to proteolysis.  

The level of the last known PTM in the FIX - β-hydroxylation of Asp64 in the EGF1 domain of FIX is slightly increased in the recombinant FIX [25], but incomplete modification of Asp64 in the natural protein clearly indicates that this PTM is not biologically important for FIX [36].  

Since the currently marketed drug of recombinant FIX is not superior to plasma-derived FIX, at least in terms of the required dose strength and duration of action, further advances in studies of recombinant FIX variants and derivatives have clinical perspectives. 

## Improvements in recombinant FIX production 

The reported secretion level of recombinant FIX by the production cell line is relatively low: ca. 30 mg/l [37]. It may be increased by 30-50% upon addition of 1 nM of methyl testosterone to the culture medium [38] or doubled after the addition of phorbol 12-myristate, 13-acetate, and calcium ionophore [39]. The appearance of undesired activated FIX in the culture medium can be controlled by decreasing the Ca ^2+^ ions concentration to 0.5 mM from 1.12 mM [40]; in another study of the same group, it was found that an increase of Ca ^2+^ ions to 1.3 mM leads to a 30% increase in the production of FIX, without a significant rise in the FIXa level [41]. 

In the early investigation of recombinant FIX from CHO cells, it was found that propeptide processing in the secreted FIX is incomplete and that FIX with an attached propeptide is inactive [42]; complete or nearly complete propeptide processing can be achieved by co-expression of the subtilisin/kexin-like convertase PACE/furin. Homologous convertase PC5 [43] may also be employed [44].  

Pro-coagulation activity of FIX requires complete gamma-carboxylation of the first 10 Glu residues in the Gla domain: last 2 residues may not be carboxylated [45]. Natural FIX is completely γ-carboxylated in all 12 residues, and in recombinant FIX the level of γ-carboxylation is reduced at last two residues, yielding an average of 11.5 Gla residues per molecule [37] and normal clotting activity (not less than 200 IU/mg). In the case of BHK host cells, the pro-coagulant activity of the secreted FIX declined for highly producing line, and over-expression of vitamin K 2,3-epoxide reductase enzyme (VKOR), which produces the cofactor for the γ-carboxylation reaction, restored the relative pro-coagulant activity to its normal level [46]. 

There are no direct investigations of the rate-limiting step in the post-translational modifications cascade for recombinant FIX, and various enhancements in the CHO enzymes levels may increase the secretion of rightly processed FIX. At least one type of modification – processing of propeptide – may be carried out in the culture medium utilizing the co-expressed soluble truncated PACE variant [37]. In the case of the homologous VKD protein – human protein C (hPC) – it was found that the rate-limiting step for recombinant hPC, expressed in human 293 cells, is N-glycosylation [47]; in the case of another VKD homologue – factor VII expressed in CHO cells – both glycosylation and γ-carboxylation limit the secretion of the product [48]. It is interesting to note that the typical secretion level of factor VII is ~5 times higher than that of FIX in industrially deployed cell lines and that the only significant difference in the post-translational modifications of these two proteins is more abundant O-glycosylation of FIX. 

CHO-derived cell lines, which are currently employed for the production of recombinant FIX, may be substituted for more productive ones. Natural FIX is produced by liver cells, and it has been established that the human hepatoma cell line HepG2 produces 1.5 times more recombinant FIX than the human kidney cell line 293 after transfection by the same retroviral vector [39]. Nonvertebrate cultured cells were also evaluated for the expression of FIX, and in a drosophila-derived SF2 cell line a 12-fold increase in functionally active FIX secretion, compared with CHO cells, was detected [49].  

## Transgenic organisms 

The milk of transgenic animals has been considered as a better source of recombinant therapeutic proteins for the last twenty years. FIX has been expressed in transgenic sheep as a fusion gene comprising the beta-lactoglobulin and FIX sequences, and small quantities of inactive FIX have been detected in the milk [50]. Higher levels of FIX, secreted in sheep milk, have been achieved using the nuclear transfer technique developed by PPL Therapeutics [51]. The producing species were created using the same technique as the one employed for Dolly the Sheep and called Molly and Polly. Similar results have been obtained for transgenic goats – 13.7 µg/l with >90% of active “gamma-glycosylated” form [52], and mice – up to 60 mg/l at 50% of biologically active FIX [53]. 

**Table 2:** Main characteristics of transgenic animals as live bioreactors and calculations of expected flock size for FIX production

Animal	Gestation, months	Maturation, months	Milk output, l*^#^	Initiation of transgene to lactation time (month)	Estimated productivity values, g*^#^,	Calculated productivity values for rFIX, g *	Reported FIX secretion values, corrected to actual concentration of active form
Mouse	0.75	1	0.0015	3-6	0.01- 0.02	0.000 045	30 mg/l [53]
Rabbit	1	5-6	2-5	7-8	20	-	-
Sheep	5	6-8	200-500	16-18	2500	5 – 12.5	25 mg/l, inactive [50]
Goat	5	6-8	600-800	16-18	4000	0.008 – 0.011	0,0137 mg/l [52]
Pig	4	6-8	200-400	15-16	1500	75 - 150	375 mg/l [55]
Cow	9	16	8,000	30-33	4,000-8,000	-	-

*- per year per doe

The most successful studies were those that used pigs [54 - 56]. Despite good theoretically predicted yields, actual factor IX production levels were moderate (summarized in Table 2). The supposed rate-limiting step in the secretion of FIX by porcine mammary gland cells is γ-carboxylation. Full specific activity (i.e. complete carboxylation) of the product was noted for these animals, producing FIX at 200 mg/l [54], and only 10-20% of normal specific activity was noted for FIX from pigs, producing at the level of 2-3 g/l [55]. Nevertheless, a viable purification process was developed for this source of under-carboxylated FIX, and the purified product, highly enriched in fully carboxylated FIX, was found to be correctly glycosylated [56] and is expected to be included in clinical trials. It should be noted that usage of milk from transgenic pigs, instead of the bioreactor harvest medium, results in approximately a 10-fold higher concentration of the target protein at the expense of a much higher level of contaminating proteins, lipids, and lack of sterility. Another protein with comparable structure complexity – antithrombin III – was expressed in goat milk at a 1-2 g/l level and purified to pharmaceutical grade with a 53% yield [57]. The consumption of FIX in the U.S. can be estimated at 2 kg/year; and world consumption, at 40 kg/year. Based on the known milk output, 40 pigs will cover the U.S. market of FIX and 800 will cover the world at the present level of expression and 50% total process yield. 

Other potential sources of biopharmaceutical proteins are the seeds and tissue of transgenic plants. At present, production of functionally active VKD proteins in transgenic plants is impossible, because the plants lack γ–carboxylases [60]. This limitation may be bypassed by co-expression in the plants of mammalian γ–carboxylases and their co-enzymes, but it is unlikely that such a complex task will be accomplished in the near future.  

Expression of inactive FIX in transgenic plants has been reported to date in transgenic tomatoes [61] and soybean seeds [62]. The expression level of FIX in tomatoes was quite low: 15.84 µg/kg of fresh fruits. In the case of soybean seeds, a very promising 800 mg/kg expression level was achieved. Both systems yielded a mature glycosylated form of the target protein.  

In some rare cases of hemophilia B (1.5–3%), large titers of neutralizing antibodies are developed by the immune system of patients in response to replacement therapy by FIX preparations [63]. These inhibitor antibodies render ordinary therapy and prophylaxis by FIX ineffective and require higher doses of FIX or immune tolerance induction (ITI) by frequent infusions of very high quantities of FIX. ITI protocols for hemophilia B last from months to years and are dangerous for patients due to the development of the nephritic syndrome and life-threatening anaphylactic reactions. At least some of the side effects in ITI treatments are caused by the excessive pro-coagulant activity of the FIX infused, and inactive variants of FIX may better serve as the immune-tolerance-inducing agent. The fusion protein of FIX and the known transmucosal carrier cholera toxin β-subunit were expressed in tobacco chloroplasts (400 mg/kg of leaf tissue) and tested as administered orally frozen leaf powder for the prevention of inhibitor antibodies formation in FIX knockout mice treated with human FIX [64]. The control animals, fed the leaf powder from untransformed tobacco, developed 2-90 Beteshda units per ml (BU/ml) of inhibitor antibodies toward human FIX, and in mice fed the leaf powder with encapsulated fusion of the toxin subunit and FIX, the inhibitor level was indistinguishable from the baseline. These data are backed by the mortality rate of treated and control mice – 10% vs. 75% after eight weekly injections of human FIX. 

## Variants of recombinant FIX 

**Fig. 3:**
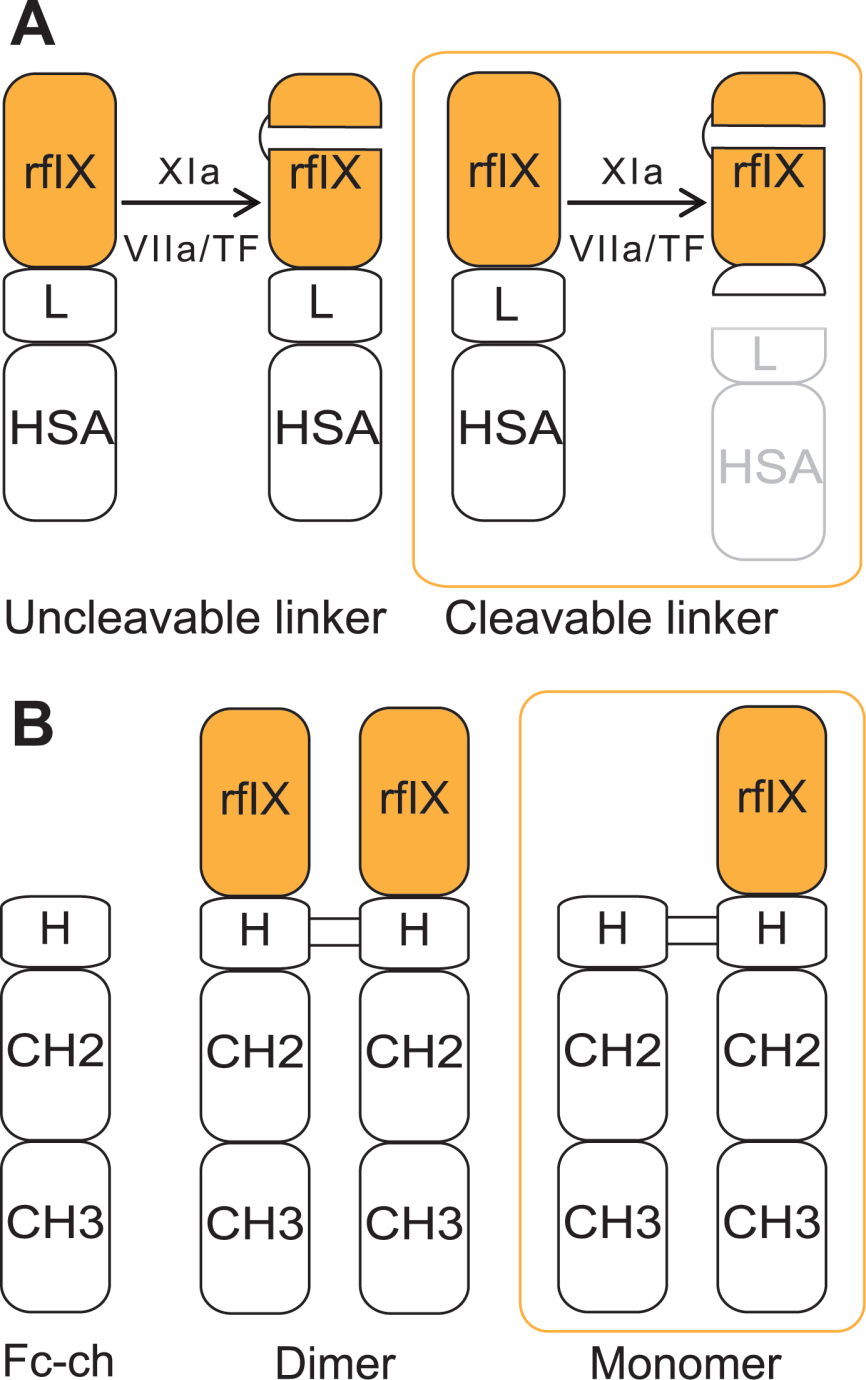
Genetic fusions of FIX. Panel A fusion of FIX and HAS, panel B fusion of FIX and Fc fragment of IgG. L – linker peptide, H – hinge region of IgG, CH2 and CH3 – second and third constant domains of IgG.

Historical use of plasma-derived FIX for hemophilia B therapy dictated the utilization of intact recombinant FIX as a drug. The exact recombinant copy of the natural FIX is expected to be nonimmunogenic for patients and perform comparably to the proven plasma-derived preparations. At the same time, current replacement therapy using FIX requires very frequent infusions of large quantities of high-priced FIX drugs. Modifications to the FIX molecule making it more active or more stable in the bloodstream may prove significantly advantageous to the patients and healthcare professionals involved. 

The specific activity of FIX in the blood coagulation assay may be increased threefold by a single point mutation, Arg338-> Ala338 [65], without affecting the tenase complex assembly. An exchange of 2 aminoacids and 2 short surface loops between FIX and factor X molecules was sufficient for the creation of a hybrid molecule with very high proteolytic activity and enzyme specificity, which is typical of factor X [66]. Further work limits the minimal set of point mutations required to 3 – Lys98, Tyr177, and Tyr94 [67]. No data on pro-coagulant activity were collected for these mutant forms of FIX. 

Since the majority of hemophiliacs suffer from the absence of factor VIII, a protein cofactor of FIX, engineered variants of FIX capable of direct activation of FX may serve as therapy for hemophilia A. They are also insensitive to inhibitor antibodies toward factor VIII. The triple mutant of FIX Val181Ile, Lys265Thr, and Ile383Val employed as the gene therapy vector successfully bypassed factor VIII and corrected the hemophilia A phenotype in mice [68]. 

The pharmacokinetic properties of FIX can be improved by genetic fusions with long-lasting plasma proteins or covalent conjugation with hydrophilic polymers. Fusion of FIX and human serum albumin has been realized through the noncleavable short linker peptide or the linker peptide cleavable by factor Xia, simultaneously with the activation of FIX [69]. The noncleavable linkers were (G) _6_ V and SS(GGS) _6_ GS, the cleavable linkers were derived from FIX and consisted of the amino-terminal activation site encompassing the amino acids 136 to 154 or 137 to 154 of mature FIX. Activation of rIX-FP with a noncleavable linker results in an activated FIXa molecule with albumin still attached to it, and activation of rIX-FP with a cleavable linker results in the release of albumin and a FIXa molecule that differs from wild-type FIXa only by a short C-terminal linker fragment derived from the FIX molecule itself ( *Fig. 3 A* ). Utilization of the cleavable linker resulted in a 10- to 30-fold increase in the specific activity of fused FIX in the coagulation assay, and the variant of albumin fusion demonstrated a significant increase in the half-life in animal models and efficacy in reducing bleeding time in FIX(-/-) mice.  

The presence of the Fc domain of immunoglobulin G enables the fusion protein to bind to the neonatal Fc receptor (FcRn), a heterodimer of the MHC class I-like protein heavy chain with beta-2-microglobulin. FcRn protects Fc-containing IgG molecules from catabolism byreversible binding to the surface of endothelial cells [70].  

Fusion of FIX and the Fc fragment expressed in human HEK-293H cells and isolated as the covalent heterodimer of the FIX-Fc chain and free Fc chain ( *Fig. 3 B* ) has acceptable specific pro-coagulant activity (ca. 50 IU/mg) and has demonstrated a significant increase in terminal half-life in many animal models [44]. The half-life in monkey was 47.3 ± 9.1 h versus 12.7 h for intact FIX. A similar 3-fold increase in half-life was recorded in phase I/II clinical trials of the FIX-Fc fusion [71].  

Group-specific attachment of PEG moiety to the N-glycans of FIX ( *Fig. 2* ) will lead to the conjugate, which will convert to natural, activated FIX, leaving the attached PEG on the released activation peptide. This kind of conjugate, with a single 40-kDa PEG group attached (N9-GP), was used in animal studies [72] and clinical trials at dose levels of 25-100 U/kg, showing a mean half-life of 93 h, 5 times higher than that of the intact FIX [73]. It is interesting to note that the incremental recovery of N9-GP was 94% higher compared with recombinant FIX, a possible indication that the PEG group may shield the FIX molecule from an undesired interaction with the cell surface or block the ability of N-glycans to mediate such an interaction. This difference in activity recovery may also be caused by the full sialation of glycans performed *in vitro* after purification of the FIX. 

Prolonged action of FIX may be achieved by various encapsulation techniques, allowing a slow release of the entrapped protein into circulation. The carrier material can be a biodegradable polymer, liposome, etc. A detailed description of this field is out of scope of the present review. A very unusual method of FIX encapsulation was recently developed for human trials [74]. Red blood cells were mixed with FIX *ex vivo* , shocked for the encapsulation of the target protein inside the cells, and injected back to the patient. The entrapped FIX slowly released from the red blood cell ghosts upon their lysis in the bloodstream.  

## Gene therapy of hemophilia B 

Simultaneously with the development of recombinant FIX protein therapeutics, gene therapy strategies for hemophilia B treatment have been developed. Protein substitution therapy has obvious limitations: treatment is not curative, and during all of the patients’ lives there remains a significant risk of bleeding episodes and chronic joint damage. Other general disadvantages of constant protein infusions are the high cost of the treatment, limited availability of the medication, low half-life of the clotting factor, and risk of neutralizing antibodies (inhibitors) formation toward the administered FIX protein. 

Both forms of hemophilia are a particularly good target for gene therapy since they are caused by a well-known single gene defect and have a broad therapeutic window: achievement of 1% of the normal plasma FIX level can prevent most patient’s risks, and a concentration of the clotting factor as high as 150%, likewise, is not expected to cause any side-effects. For gene therapy, the therapeutic level of the expressed FIX is usually considered as 5-10% of the normal plasma level; in this case further protein injections might be avoided. A low (under-therapeutic) expression level of FIX may still be enough for immune tolerance induction in patients suffering from the inhibitory form of the disease.  

Transfer of the FIX gene *in vivo* is possible even by the naked plasmid DNA, as has been shown in animal models. Hydrodynamic injection of expression plasmid containing human FIX cDNA and the hepatic control region was sufficient to achieve therapeutic levels of FIX in deficient mice for 210 days [75]. The delivery technique employed – injection of 50 µg of the plasmid in 2 ml of solution in 5-8 sec into the tail vein – is definitely unsuitable for human therapy, and a significant modification of hydrodynamic injection should be invented before clinical studies can take place.  

Target cDNA may be delivered more efficiently by chromosome-integrating viral vectors of retroviral (RV) or lentiviral (LV) origin or by predominantly episomal adenoviruses (AV) or adeno-assoсiated viruses (AAV). 

Historically, RV particles had been used first for the transduction *ex vivo* of fibroblasts from model animals by FIX cDNA and subsequent re-implantation of the modified cells. A low level of human FIX was detected in the plasma of the treated animals [76]. A very small percentage of the animals with re-implanted transduced fibroblasts test positive for FIX production, but the effect remains stable for more than 600 days on the rabbit model [77]. A phase I clinical trial for hemophilia B was conducted with autologous skin fibroblasts transduced *ex vivo* with FIX-encoding γ-retroviral vectors [78] and resulted in a transient, moderate increase in the FIX plasma level in two patients.  

Lentiviral vectors, in contrast to γ-retroviral vectors, are able to transduce hepatocytes of the adult liver *in vivo* . Therapeutic levels of FIX have been achieved (transiently) in adult hemophilic mice, following intravenous injection of LV [79]. LV are also able to effectively transduce spleen antigen-presenting cells (APC), leading to an immune response against circulating transgene proteins [80]. This unwanted ability of LV may be diminished by restricting transgene expression to certain cell types by utilizing tissue-specific promoter sequences and by co-expression of microRNA’s, eliminating off-target expression. Long-term FIX expression in mice using a hepatocyte-specific promoter and hematopoietic cells-specific microRNA miR-142-3p has resulted in a more than 10% level of circulated FIX for 280 days in hemophilia B model mice [81]. No antibodies toward FIX were detected, and all animals survived after a challenge by tail-clip.  

Expression of FIX may also be restricted to hematopoietic cells to ensure better availability of the target protein to the sites of its action. Integrin alpha II b promoter-bearing LV constructs, expression-targeted to megakariocytes, were used in hemophilia B mice models and showed promising results: accumulation of FIX in the alpha-granules of platelets and release after activation [82], and phenotype correction was proven by full survivability after tail-clip. 

Common to all integrating viral vectors, including RV and LV, are safety concerns of insertional mutagenesis and oncogene activation after vector integration [80]; so vectors with episomic persistence, bearing FIX transgene, are attracting much more attention. High-capacity adenoviral vectors (HCAV) with episomic persistence, bearing no viral genes, are known to trigger a reduced immune response, and the use of a tissue-specific promoter (e.g. hepatic) can further diminish the response, therefore prolonging the gene expression period [83]. HCAV with a liver-specific promoter has yielded therapeutic expression levels of IX with limited toxicity in hemophilic mice [84] and hemophilic dogs [85, 86], and yet a gradual decline in transgene expression was observed. Inhibitor antibodies, as well as hematologic and hepatic toxicities, were detected in animals injected at high vector doses [86], limiting the expression period to 446-604 days in dogs.  

Adeno-associated viruses are believed to be better carriers of target genes at the expense of limited packaging capacity, not exceeding 4.7 k.b.p. They are nonpathogenic, replication-deficient, and have a very low probability of chromosomal integration. [87] 

AAV particles for gene therapy studies can be manufactured by the GMP-compliant process [88]. In most clinical trials with AAV vectors intramuscular injection or portal vein infusion routes have been used, directing viral particles to skeletal muscle cells or hepatocytes.  

Long-term FIX expression has been achieved following muscle-directed gene transfer by the AAV2-FIX vector in hemophilia B dogs with a missense mutation [89]. Inhibitor antibodies development depended on the nature of the FIX gene defect in the treated animals – dogs with missense mutation in the FIX gene developed virtually no inhibitors [89], and dogs with premature stop codon and unstable mRNA in the FIX gene developed a significant level of the inhibitor [90] that correlated with the AAV dose used [91]. A phase I clinical trial for intramuscular injection of AAV2-FIX vectors was performed in hemophilia B patients with missense mutations using a limited vector dose per site [92-94]. The treatment proved safe but ineffective: the achieved FIX levels were below the therapeutic level.  

Utilization of a more invasive procedure aimed at delivering AAV particles to the liver resulted in FIX expression without inhibitor development in normal and hemophilic mice, hemophilia B dogs, and nonhuman primates [87, 95-99]. An 8-year study in inhibitor-prone null mutation hemophilia B dogs treated with liver-directed AAV2-FIX demonstrated long-term hemophilia correction without inhibitor development [100].  

A phase I clinical trial was conducted for hemophilia B patients with intrahepatic infusion of AAV2 vectors encoding FIX and a liver-specific promoter [101]. FIX levels of up to 10% were achieved, but the transgene expression period was no longer than 6 weeks, most likely the result of an immune response toward capsid components. It has been suggested that the rapid elimination of the transduced viruses could be caused by the pre-existing immune response to wild-type AAV2 viruses, which are common in the population [101, 102]. Other serotypes of AAV, namely AAV8 and AAV9, both having a liver tropism and less common in human population [103], have been tested on model animals. AAV8 has been found to be a more effective carrier of the FIX gene than AAV2 in mice and dog models [104], [105] and has demonstrated long-term (up to 5 years) safety and efficacy in nonhuman primates [106], with a constant therapeutic level of transduced FIX in the optimal vector dose group. Phase I clinical trials are being conducted for the AAV8-FIX delivered by peripheral vein infusion. The expected immune reactions were diminished by a several-weeks course of prednisolone; the levels of FIX achieved have been in the range of 1% to 8% of normal values in the six patients treated. Two of them cut back infusions of FIX, and four have gone off infusions [107].

## Conclusion 

Despite a sustained research effort, existing therapy for hemophilia B relies mainly on the infusion of FIX, with no significant justification for the bias for plasma-derived or recombinant protein. Modification of the FIX molecule by domain fusions or conjugation with PEG may decrease the frequency of infusions, but rather will change the cost and overall safety of the treatment. Gene therapy by FIX cDNA in viral vectors is promising for a significant proportion of patients. It has the potential to diminish the need for transfusions for the majority and completely eliminate that need for some. Hopefully, the straight increase in the production of recombinant FIX achieved through the adoption of biosimilars produced using more productive cell lines and transgenic animals will yield benefits to the world’s population of hemophilia B patients in the years to come.  

## Abbreviations

FIX: Factor IX
rFIX: recombinant factor IX
rhFIX - recombinant human factor IX; FIXa - activated FIX; pdFIX - plasma-derived factor IX; PTM - posttranslational modification; VKD - vitamin K dependent; IU: international unit
EGF: epidermal growth factor

## References

[1] Zhong D., Bajaj M.S., Schmidt A.E., Bajaj S.P. (2002). J. Biol. Chem..

[2] Wright I.S. (1962). Can. Med. Assoc. J..

[3] Howard E.L., Becker K.C., Rusconi C.P., Becker R.C. (2007). Arterioscler. Thromb. Vasc. Biol..

[4] Green P.M., Giannelli F., Sommer S.S., Poon M.-C., Ludwig M., Schwaab R., Reitsma P.H., Goossens M., Yoshioka A., Figueiredo M.S. www.kcl.ac.uk/ip/petergreen/haemBdatabase.html..

[5] Rogaev E.I., Grigorenko A.P., Faskhutdinova G., Kittler E.L., Moliaka Y.K. (2009). Science..

[6] Reitsma P.H., Bertina R.M., Ploos van Amstel J.K., Riemens A., Briet E. (1988). Blood..

[7] Evatt B.L., Black C., Batorova A., Street A., Srivastava A. (2004). Haemophilia..

[8] Tabor E. (1999). Transfusion..

[9] Kim H.C., McMillan C.W., White G.C., Bergman G.E., Horton M.W., Saidi P. (1992). Blood..

[10] Ludlam C.A., Powderly W.G., Bozzette S., Diamond M., Koerper M.A., Kulkarni R., Ritchie B., Siegel J., Simmonds P., Stanley S. (2006). Lancet..

[11] Kurachi K., Davie E.W. (1982). Proc. Natl. Acad. Sci. USA..

[12] Anson D.S., Austen D.E., Brownlee G.G. (1985). Nature..

[13] de la Salle H., Altenburger W., Elkaim R., Dott K., Dieterle A., Drillien R., Cazenave J.P., Tolstoshev P., Lecocq J.P. (1985). Nature..

[14] Busby S., Kumar A., Joseph M., Halfpap L., Insley M., Berkner K., Kurachi K., Woodbury R. (1985). Nature..

[15] Kaufman R.J., Wasley L.C., Furie B.C., Furie B., Shoemaker C.B. (1986). J. Biol. Chem..

[16] Harrison S., Adamson S., Bonam D., Brodeur S., Charlebois T., Clancy B., Costigan R., Drapeau D., Hamilton M., Hanley K. (1998). Semin. Hematol..

[17] Bush L., Webb C., Bartlett L., Burnett B. (1998). Semin. Hematol..

[18] Lambert T., Recht M., Valentino L.A., Powell J.S., Udata C., Sullivan S.T., Roth D.A. (2007). Haemophilia..

[19] White G., Shapiro A., Ragni M., Garzone P., Goodfellow J., Tubridy K., Courter S. (1998). Semin. Hematol..

[20] Rup B. (2002). Dev. Biol. (Basel)..

[21] Bond M., Jankowski M., Patel H., Karnik S., Strang A., Xu B., Rouse J., Koza S., Letwin B., Steckert J. (1998). Semin. Hematol..

[22] Ewenstein B.M., Joist J.H., Shapiro A.D., Hofstra T.C., Leissinger C.A., Seremetis S.V., Broder M., Mueller-Velten G., Schwartz B.A. (2002). Transfusion..

[23] Bjorkman S. (2011). Haemophilia..

[24] Rouse J.C., McClellan J.E., Patel H.K., Jankowski M.A., Porter T.J. (2005). Methods Mol. Biol..

[25] Derian C.K., VanDusen W., Przysiecki C.T., Walsh P.N., Berkner K.L., Kaufman R.J., Friedman P.A. (1989). J. Biol. Chem..

[26] Spitzer S.G., Kuppuswamy M.N., Saini R., Kasper C.K., Birktoft J.J., Bajaj S.P. (1990). Blood..

[27] Rees D.J., Jones I.M., Handford P.A., Walter S.J., Esnouf M.P., Smith K.J., Brownlee G.G. (1988). EMBO J..

[28] Ahmad S.S., Rawala R., Cheung W.F., Stafford D.W., Walsh P.N. (1995). Biochem. J..

[29] Chang Y.J., Wu H.L., Hamaguchi N., Hsu Y.C., Lin S.W. (2002). J. Biol. Chem..

[30] Larson P.J., Stanfield-Oakley S.A., VanDusen W.J., Kasper C.K., Smith K.J., Monroe D.M., High K.A. (1996). J. Biol. Chem..

[31] Harris R.J., van Halbeek H., Glushka J., Basa L.J., Ling V.T., Smith K.J., Spellman M.W. (1993). Biochemistry..

[32] Agarwala K.L., Kawabata S., Takao T., Murata H., Shimonishi Y., Nishimura H., Iwanaga S. (1994). Biochemistry..

[33] Kaufman R.J. (1998). Thromb. Haemost..

[34] Makino Y., Omichi K., Kuraya N., Ogawa H., Nishimura H., Iwanaga S., Hase S. (2000). J. Biochem..

[35] Bharadwaj D., Harris R.J., Kisiel W., Smith K.J. (1995). J. Biol. Chem..

[36] Sunnerhagen M.S., Persson E., Dahlqvist I., Drakenberg T., Stenflo J., Mayhew M., Robin M., Handford P., Tilley J.W., Campbell I.D. (1993). J. Biol. Chem..

[37] McGrath B.M., Walsh G. (2005). Directory of therapeutic enzymes. N.Y.: Taylor & Francis,.

[38] Dadehbeigi N., Ostad S.N., Faramarzi M.A., Ghahremani M.H. (2008). Biotechnol. Lett..

[39] De Castilho Fernandes A., Fontes A., Gonsales N., Swiech K., Picanco-Castro V., Faca S., Covas D. (2011). Biotechnol. Appl. Biochem..

[40] Kim W.H., Kim J.S., Yoon Y., Lee G.M. (2009). J. Biotechnol..

[41] Lim I., Kim J.-S., Lee G., Choi M., Yoon Y. (2010). Cells and Culture / Ed. Noll T. Amsterdam: Springer
Netherlands,.

[42] Wasley L.C., Rehemtulla A., Bristol J.A., Kaufman R.J. (1993). J. Biol. Chem..

[43] Lusson J., Vieau D., Hamelin J., Day R., Chretien M., Seidah N.G. (1993). Proc. Natl. Acad. Sci. USA..

[44] Peters R.T., Low S.C., Kamphaus G.D., Dumont J.A., Amari J.V., Lu Q., Zarbis-Papastoitsis G., Reidy T.J., Merricks E.P., Nichols T.C. (2010). Blood..

[45] Gillis S., Furie B.C., Furie B., Patel H., Huberty M.C., Switzer M., Foster W.B., Scoble H.A., Bond M.D. (1997). Protein Sci..

[46] Wajih N., Hutson S.M., Owen J., Wallin R. (2005). J. Biol. Chem..

[47] McClure D.B., Walls J.D., Grinnell B.W. (1992). J. Biol. Chem..

[48] Bolt G., Steenstrup T.D., Kristensen C. (2007). Thromb. Haemost..

[49] Vatandoost J., Zomorodipour A., Sadeghizadeh M., Aliyari R., Bos M.H., Ataei F. (2012). Biotechnol. Prog..

[50] Clark A.J., Ali S., Archibald A.L., Bessos H., Brown P., Harris S., McClenaghan M., Prowse C., Simons J.P., Whitelaw C.B. (1989). Genome..

[51] Schnieke A.E., Kind A.J., Ritchie W.A., Mycock K., Scott A.R., Ritchie M., Wilmut I., Colman A., Campbell K.H. (1997). Science..

[52] Zhang K., Wang H., Bao Y., Lu D., Xue J., Qiu X., Huang S., Huang Y., Li B., Li H. (1997). Chinese Sci. Bull..

[53] Yull F., Harold G., Wallace R., Cowper A., Percy J., Cottingham I., Clark A.J. (1995). Proc. Natl. Acad. Sci. USA..

[54] van Cott K.E., Butler S.P., Russell C.G., Subramanian A., Lubon H., Gwazdauskas F.C., Knight J., Drohan W.N., Velander W.H. (1999). Genet. Anal..

[55] Lindsay M., Gil G.C., Cadiz A., Velander W.H., Zhang C., van Cott K.E. (2004). J. Chromatogr. A..

[56] Gil G.C., Velander W.H., van Cott K.E. (2008). Glycobiology..

[57] Edmunds T., van Patten S.M., Pollock J., Hanson E., Bernasconi R., Higgins E., Manavalan P., Ziomek C., Meade H., McPherson J.M. (1998). Blood..

[58] Dove A. (2000). Nat. Biotechnol..

[59] Panno J. (2004). Animal Cloning: The Science of Nuclear Transfer. N.Y.: Facts on
File,.

[60] Gomord V., Faye L. (2004). Curr. Opin. Plant Biol..

[61] Zhang H., Zhao L., Chen Y., Cui L., Ren W., Tang K. (2007). Biotechnol. Appl. Biochem..

[62] Cunha N.B., Murad A.M., Ramos G.L., Maranhao A.Q., Brigido M.M., Araujo A.C., Lacorte C., Aragao F.J., Covas D.T., Fontes A.M. (2011). Transgenic Res..

[63] DiMichele D. (2007). Br. J. Haematol..

[64] Verma D., Moghimi B., LoDuca P.A., Singh H.D., Hoffman B.E., Herzog R.W., Daniell H. (2010). Proc. Natl. Acad. Sci. USA..

[65] Chang J., Jin J., Lollar P., Bode W., Brandstetter H., Hamaguchi N., Straight D.L., Stafford D.W. (1998). J. Biol. Chem..

[66] Hopfner K.P., Brandstetter H., Karcher A., Kopetzki E., Huber R., Engh R.A., Bode W. (1997). EMBO J..

[67] Sichler K., Kopetzki E., Huber R., Bode W., Hopfner K.P., Brandstetter H. (2003). J. Biol. Chem..

[68] Milanov P., Ivanciu L., Abriss D., Quade-Lyssy P., Miesbach W., Alesci S., Tonn T., Grez M., Seifried E., Schuttrumpf J. (2012). Blood..

[69] Metzner H.J., Weimer T., Kronthaler U., Lang W., Schulte S. (2009). Thromb. Haemost..

[70] Dumont J.A., Low S.C., Peters R.T., Bitonti A.J. (2006). BioDrugs..

[71] Shapiro A.D., Ragni M., Valentino L.A., Key N.S., Josephson N., Powell J., Cheng G., Tubridy K.L., Peters R., Dumont J. (2010). Haemophilia..

[72] Ostergaard H., Bjelke J.R., Hansen L., Petersen L.C., Pedersen A.A., Elm T., Moller F., Hermit M.B., Holm P.K., Krogh T.N. (2011). Blood..

[73] Negrier C., Knobe K., Tiede A., Giangrande P., Moss J. (2011). Blood..

[74] Sinauridze E.I., Vuimo T.A., Kulikova E.V., Shmyrev I.I., Ataullakhanov F.I. (2010). Med. Sci. Monit..

[75] Kim H.S., Kim J.C., Lee Y.K., Kim J.S., Park Y.S. (2011). J. Gene Med..

[76] Palmer T.D., Thompson A.R., Miller A.D. (1989). Blood..

[77] Chen L., Nelson D.M., Zheng Z., Morgan R.A. (1998). Hum. Gene Ther..

[78] Qiu X., Lu D., Zhou J., Wang J., Yang J., Meng P., Hsueh J.L. (1996). Chin. Med. J. (Engl.)..

[79] Tsui L.V., Kelly M., Zayek N., Rojas V., Ho K., Ge Y., Moskalenko M., Mondesire J., Davis J., Roey M.V. (2002). Nat. Biotechnol..

[80] Petrus I., Chuah M., van den Driessche T. (2010). J. Gene Med..

[81] Brown B.D., Cantore A., Annoni A., Sergi L.S., Lombardo A., Della Valle P., D'Angelo A., Naldini L. (2007). Blood..

[82] Zhang G., Shi Q., Fahs S.A., Kuether E.L., Walsh C.E., Montgomery R.R. Blood. V..

[83] Pastore L., Morral N., Zhou H., Garcia R., Parks R.J., Kochanek S., Graham F.L., Lee B., Beaudet A.L. (1999). Hum. Gene Ther..

[84] Ehrhardt A., Kay M.A. (2002). Blood..

[85] Ehrhardt A., Xu H., Dillow A.M., Bellinger D.A., Nichols T.C., Kay M.A. (2003). Blood..

[86] Brunetti-Pierri N., Nichols T.C., McCorquodale S., Merricks E., Palmer D.J., Beaudet A.L., Ng P. (2005). Hum. Gene Ther..

[87] Snyder R.O., Miao C.H., Patijn G.A., Spratt S.K., Danos O., Nagy D., Gown A.M., Winther B., Meuse L., Cohen L.K. (1997). Nat. Genet..

[88] Allay J.A., Sleep S., Long S., Tillman D.M., Clark R., Carney G., Fagone P., McIntosh J.H., Nienhuis A.W., Davidoff A.M. Hum. Gene Ther. V..

[89] Herzog R.W., Yang E.Y., Couto L.B., Hagstrom J.N., Elwell D., Fields P.A., Burton M., Bellinger D.A., Read M.S., Brinkhous K.M. (1999). Nat. Med..

[90] Herzog R.W., Mount J.D., Arruda V.R., High K.A., Lothrop C.D. (2001). Mol. Ther..

[91] Herzog R.W., Fields P.A., Arruda V.R., Brubaker J.O., Armstrong E., McClintock D., Bellinger D.A., Couto L.B., Nichols T.C., High K.A. (2002). Hum. Gene Ther..

[92] Kay M.A., Manno C.S., Ragni M.V., Larson P.J., Couto L.B., McClelland A., Glader B., Chew A.J., Tai S.J., Herzog R.W. (2000). Nat. Genet..

[93] Manno C.S., Chew A.J., Hutchison S., Larson P.J., Herzog R.W., Arruda V.R., Tai S.J., Ragni M.V., Thompson A., Ozelo M. (2003). Blood..

[94] Jiang H., Pierce G.F., Ozelo M.C., de Paula E.V., Vargas J.A., Smith P., Sommer J., Luk A., Manno C.S., High K.A. (2006). Mol. Ther..

[95] Snyder R.O., Miao C., Meuse L., Tubb J., Donahue B.A., Lin H.F., Stafford D.W., Patel S., Thompson A.R., Nichols T. (1999). Nat. Med..

[96] Nathwani A.C., Davidoff A.M., Hanawa H., Hu Y., Hoffer F.A., Nikanorov A., Slaughter C., Ng C.Y., Zhou J., Lozier J.N. (2002). Blood..

[97] Mount J.D., Herzog R.W., Tillson D.M., Goodman S.A., Robinson N., McCleland M.L., Bellinger D., Nichols T.C., Arruda V.R., Lothrop C.D. (2002). Blood..

[98] Wang L., Nichols T.C., Read M.S., Bellinger D.A., Verma I.M. (2000). Mol. Ther..

[99] Wang L., Takabe K., Bidlingmaier S.M., Ill C.R., Verma I.M. (1999). Proc. Natl. Acad. Sci. USA..

[100] Niemeyer G.P., Herzog R.W., Mount J., Arruda V.R., Tillson D.M., Hathcock J., van Ginkel F.W., High K.A., Lothrop C.D. (2009). Blood..

[101] Manno C.S., Pierce G.F., Arruda V.R., Glader B., Ragni M., Rasko J.J., Ozelo M.C., Hoots K., Blatt P., Konkle B. (2006). Nat. Med..

[102] Mingozzi F., Maus M.V., Hui D.J., Sabatino D.E., Murphy S.L., Rasko J.E., Ragni M.V., Manno C.S., Sommer J., Jiang H. (2007). Nat. Med..

[103] van den Driessche T., Thorrez L., Acosta-Sanchez A., Petrus I., Wang L., Ma L., Waelle D.E., Iwasaki Y., Gillijns V., Wilson J.M. (2007). J. Thromb. Haemost..

[104] Cooper M., Nayak S., Hoffman B.E., Terhorst C., Cao O., Herzog R.W. (2009). Hum. Gene Ther..

[105] Wang L., Calcedo R., Nichols T.C., Bellinger D.A., Dillow A., Verma I.M., Wilson J.M. (2005). Blood..

[106] Nathwani A.C., Rosales C., McIntosh J., Rastegarlari G., Nathwani D., Raj D., Nawathe S., Waddington S.N., Bronson R., Jackson S. (2011). Mol. Ther..

[107] Nathwani A.C., Tuddenham E.G., Rangarajan S., Rosales C., McIntosh J., Linch D.C., Chowdary P., Riddell A., Pie A.J., Harrington C. (2011). N. Engl. J. Med..

